# Supplementary material for: The Magnetosome Protein, Mms6 from Magnetospirillum magneticum Strain AMB-1, Is a Lipid-Activated Ferric Reductase
Source: Int J Mol Sci. 2022 Sep 7;23(18):10305. doi: 10.3390/ijms231810305 (PMC9499114; doi:10.3390/ijms231810305)
Supplement: Supplementary file 1 [file ijms-23-10305-s001.zip › ijms-1855886-supplementary.pdf]

## Supplementary Materials
# The Magnetosome Protein, Mms6 from *Magnetospirillum magneticum* strain AMB-1, is a Lipid-Activated Ferric Reductase

Dilini Singappuli Arachchige,[†] Shuren Feng,[$¶] Lijun Wang,[$¶] Pierre E. Palo[$], Samuel O. Shobade[$¶], Michelle Thomas[$] and Marit Nilsen-Hamilton,[†$¶*]

**Table S1. List of protein sequences used in this work**

| protein name | Sequence |
|---|---|
| Mms6 | VGGTIWTGKGLGLGLGLGLGAWGPIILGVVGAGAVYAYMKSRDIESAQSDEEVELRDALA |
| m1Mms6 | VGGTIWTGKGLGLGLGLGLGAWGPIILGVVGAGAVYAYADSEDIESAQSRRKVELEDALA |
| m2Mms6 | VGGTIWTGKGLGLGLGLGLGAWGPIILGVVGAGAVYAYAKDRSIDEAQESDSVELREALA |
| m3Mms6 | VGGTIWTGKGLGLGLGLGLGAWGPIILGVVGAGAVYAYAQSLERAEDEDADISAVEKLSR |
| C21Mms6 | KSRDIESAQSDEEVELRDALA |
| GLtoGA | VGGTIWTGKGAGAGAGAGAGAWGPIILGVVGAGAVYAYMKSRDIESAQSDEEVELRDALA |
| W103A | VGGTIATGKGLGLGLGLGLGAWGPIILGVVGAGAVYAYMKSRDIESAQSDEEVELRDALA |
| W119A | VGGTIWTGKGLGLGLGLGLGAAGPIILGVVGAGAVYAYMKSRDIESAQSDEEVELRDALA |
| S138A | VGGTIWTGKGLGLGLGLGLGAWGPIILGVVGAGAVYAYMKARDIESAQSDEEVELRDALA |
| D140A | VGGTIWTGKGLGLGLGLGLGAWGPIILGVVGAGAVYAYMKSRAIESAQSDEEVELRDALA |
| S146A | VGGTIWTGKGLGLGLGLGLGAWGPIILGVVGAGAVYAYMKSRDIESAQADEEVELRDALA |
| D147A | VGGTIWTGKGLGLGLGLGLGAWGPIILGVVGAGAVYAYMKSRDIESAQSAEEVELRDALA |
| E148A | VGGTIWTGKGLGLGLGLGLGAWGPIILGVVGAGAVYAYMKSRDIESAQSDAEVELRDALA |
| E149A | VGGTIWTGKGLGLGLGLGLGAWGPIILGVVGAGAVYAYMKSRDIESAQSDEAVELRDALA |
| D154A | VGGTIWTGKGLGLGLGLGLGAWGPIILGVVGAGAVYAYMKSRDIESAQSDEEVELRAALA |
| S146E148A | VGGTIWTGKGLGLGLGLGLGAWGPIILGVVGAGAVYAYMKSRDIESAQADAEVELRDALA |
| Histidine tag: | MGGSHHHHHHGMASMTGGQQMGRDLYDDDDKDPTLGGHM |
| His-Ubi-Mms6 | MGHHHHHHHHHHSSGHIDDDAKHMQIFVKTLTGKTITLEVESSDTIDNVKSKI QDKEGIPPDQQRLIFAGKQLEDGRTLSDYNIQKESTLHLVLRLRGGHIETLYFQGHMPSTLTPR GGTIWTGKGLGLGLGLGLGAWGPIILGVVGAGAVYAYMKSRDIESAQSDEEVELRDALA |

**Legend:** All proteins, except for C21Mms6, which was chemically synthesized, were produced as fusion proteins with an N-terminal histidine tag. All except Hi-Ubi-Mms6 included the histidine tag shown in the second to last row. His-Ubi-Mms6 is shown in the last row with its histidine tag.

**Table S2. Summary of kcat determinations**

| Operator | Conditions | | $10^5$ x $k_{cat}$ | Individual preparations ($10^5$ x $k_{cat}$) | | | | | | | |
|---|---|---|---|---|---|---|---|---|---|---|---|
| Individual | prep | date | /sec | 1178 | 1628 | 1629 | 1633 | 1634 | 1639 | 14611 | 14619 |
| 2 | 1178 | 2/15/2014 | 2.6 | 2.6 | | | | | | | |
| 1 | 1628 | 8/28/2013 | 1.2 | | 1.2 | | | | | | |
| 2 | 1633 | 12/14/2013 | 1.8 | | | | 1.8 | | | | |
| 2 | 1634 | 12/15/2013 | 2.2 | | | | | 2.2 | | | |
| 2 | 1629 | 12/16/2013 | 2.3 | | | 2.3 | | | | | |
| 2 | 1629 | 12/18/2013 | 2.0 | | | 2.0 | | | | | |
| 2 | 1633 | 12/18/2013 | 2.4 | | | | 2.4 | | | | |
| 2 | 1634 | 12/18/2013 | 3.3 | | | | | 3.3 | | | |
| 2 | 1629 | 1/18/2014 | 1.7 | | | 1.7 | | | | | |
| 2 | 1633 | 1/18/2014 | 0.62 | | | | 6.2 | | | | |
| 2 | 1639 | 6/27/2014 | 0.30 | | | | | | 3.0 | | |
| 3 | 14611 | 10/5/2020 | 1.8 | | | | | | | 1.8 | |
| 3 | 14611 | 10/14/2020 | 1.4 | | | | | | | 1.4 | |
| 3 | 14619 | 6/21/2022 | 16 | | | | | | | | 16 |
| 3 | 14619 | 6/21/2022 | 15 | | | | | | | | 15 |
| 3 | 14611 | 6/24/2022 | 343 | | | | | | | 343 | |
| 3 | 14611 | 6/24/2022 | 342 | | | | | | | 342 | |
| 3 | 14611 | 6/27/2022 | 5.9 | | | | | | | 5.9 | |
| 3 | 14611 | 6/27/2022 | 11 | | | | | | | 11 | |
| 3 | 14611 | 7/1/2022 | 14 | | | | | | | 14 | |
| 3 | 14611 | 7/1/2022 | 9.4 | | | | | | | 9.4 | |
| 3 | 14611 | 7/1/2022 | 0.60 | | | | | | | 0.60 | |
| 3 | 14611 | 7/1/2022 | 0.44 | | | | | | | 4.4 | |

| | $10^5$ x kcat averages | | $10^5$ x kcat | | | | | | | |
|---|---|---|---|---|---|---|---|---|---|---|
| | All values | minus highest and lowest values | Individual preparations | | | | | | | |
| Average | 30 | 4.8 | 2.6 | 1.2 | 2.0 | 1.6 | 2.7 | 0.30 | 69 | 15 |
| standard deviation | 99 | 5.2 | | | 0.27 | 0.88 | 0.82 | | 144 | 0.86 |
| coefficient of variation | 326% | 109% | | | 14% | 56% | 30% | | 209% | 6% |
| Median | 2.3 | 2.2 | | | | | | | | |
| Minimum | 0.30 | 0.44 | | | | | | | | |
| Maximum | 343 | 16 | | | | | | | | |
| N | 23 | 20 | 1 | 1 | 3 | 3 | 2 | 1 | 10 | 2 |

**LEGEND:** The results of $k_{cat}$ measurements for a range of Mms6 preparations by three different people over a period of 9 years.

**Table S3. Homologs identified by TM-Align**

| PDB | Homologs identified by TM-Align | Microbial source |
|---|---|---|
| 3V2H | D-beta-hydroxybutyrate dehydrogenase | *Sinorhizobium meliloti* |
| 3GVI | Lactate/malate dehydrogenase in complex with ADP | *Brucella melitensis* |
| 3FG2 | Ferredoxin Reductase for the CYP199A2 System | *Rhodopseudomonas palustris* |
| 7DNM | AgCarB2-C2 complex | *Arthrobacter globiformis* |
| 5LC5 | Mammalian respiratory complex I, class2 | *Bos taurus* |
| 6AGS | L310R/Q401C mutant of malic enzyme | *Escherichia coli* |
| 2VCF | Cytosolic ascorbate peroxidase with isoniazid (INH) | *Glycine max* |
| 6T15 | III2-IV(5B)1 respiratory supercomplex | Saccharomyces cerevisiae |
| 1D4C | Flavocytochrome c fumarate reductase, uncomplexed | Shewanella putrefaciens strain mr-2 |
| 3EF6 | Toluene 2,3-dioxygenase reductase | *Pseudomonas putida* |
| 1YCG | Nitric oxide reductase | *Moorella thermoacetica FprA* |
| 3KD9 | Pyridine nucleotide disulfide oxidoreductase | *Pyrococcus horikoshii* |

**LEGEND:** Enzymes identified by TM-Align as structural homologs of Mms6. The top three structures were used by I-TASSER as the basis of structural prediction.

## Table S4. Summary of reported kinetic parameters

| Bacterium | MW | pH optimum | electron donor | cofactor | electron acceptor ($K_d$) | | Specific activity (nmole/min/mg) | Citations |
|---|---|---|---|---|---|---|---|---|
| | | | NADH or NADPH | FMN vs. FAD | | | | |
| Mms6, M. magnetotacticum | | | NADH preferred | FAD | | Fe-Citrate with FAD, NADH (76) | Fe-citrate (5.7) | this work |
| Mms6, M. magnetotacticum in bicelles | | | | | | | Fe-citrate (20) | this work |
| Archaeoglobus fulgidus | 18 | 7 | both | both | Fe-EDTA with NADH (61), with NADPH (80); flavin not specified | Fe-citrate (20.6) | | 56 |
| Azotobacter vinelandii | 44.6 and 69 | 7.5 | preferred (16) | FMN preferred | Fe-Citrate with NADH, FMN (10) | Fe-citrate (21) | Fe-citrate (197) | 57 |
| Bacillus subtilis | N/R | | NADPH | FMN | Fe-HBA with NADPH, FMN (55) | Fe-citrate (197) | Fe-DCIP (3.8) | 58 |
| Bacillus subtilis | 13 | | NADPH (78) with oxygen as acceptor | FMN (12.5) with oxygen as acceptor | Fe-DCIP (385) | Fe-DCIP (3.8) | | 41 |
| E. coli | 26 | | both equal | both but FMN preferred | | | | 53 |
| E. coli | 26 | | NADH (9), NADPH (30) | FMN (1.5), FAD (1.9) | | | | 53 |
| *L. pneumophila* | 38 | | NADH (11), NADPH (9) | | | | Fe-citrate (7) | 59 |
| *M. magnetotacticum* | 36 | 7 | NADH (1.3) NADPH (119) | FMN (0.035) | Fe-Citrate with FMN, NADH (15) | Fe-citrate (3111) | | 60 |
| *R. sphaeroides* | 32 | 7.1 | NADH (18) not NADPH | FMN (3.2), FAD (14.1) | Fe-Citrate with FMN, NADH (8.3) | | | 61 |
| *R. sphaeroides* | 41 | | NADH (20) not NADPH | | Fe-Citrate with FMN, NADH (4.2) | Fe-citrate (1300), Fe-EDTA (13100) | | 61 |
| *Acidithiobacillus ferrooxidans* | 28 | 6.5 | NADPH (76) not NADH | FMN | Fe-EDTA with NADPH, FMN (30) | Fe-citrate (1300), ferric-EDTA (13000) | | 62 |
| Paracoccus denitrificans | 19 | | NADH (5.5) not NADPH | FMN (20) | Fe-NLA with FMN, NADH (17) | Fe-EDTA (0.005); Fe-NLA (7.6) | | 63 |
| Paracoccus denitrificans | 18 | | NADH (2.6) | FAD | | Fe-NLA (3.1), Fe-EDTA (0.16), Fe-citrate (0.08) | Fe-NLA (4.8) | 63 |
| Pseudomonas putida | 29.5 | | NADPH (6.3) | both | Fe-EDTA with NADH, FAD (19), with NADH, FMN (8.7), with NADPH, FMN or FAD (~7); Fe-citrate with NADH, FAD (12), with FAD, NADPH (6.4) | | | 64 |
| Pseudomonas putida | 29.4 | | NADH (2.4) | both | Fe-EDTA with NADH, FAD (13), with NADH, FMN (6.5), with NADPH, FMN or FAD (~15): Fe-citrate with NADH, FAD (6.2), with FAD, NADPH (12) | | | 64 |
| Magnetospirillum gryphiswaldense | 16 | 6.5 | NADH not NADPH | FMN (0.24) | Fe-citrate with FAD, NADPH (45) | | | 65 |
| G. sulfurreducens | 78, 87 | 5.5 | NADPH (25) not NADH | none added, a flavin is associated with the enzyme | Fe-NLA with NADPH (1000) | Fe-NLA with NADPH (65000) | Fe-NLA (65000) | 66 |

| **Statistical parameters** | **MW (KDa)** | **pH optimum** | **electron donor ($K_d$)** | **Cofactor ($K_d$)** | **Fe(III)-EDTA ($K_M$)** | **Fe(III)-citrate ($K_M$)** | **Specific activity (nmole/min/mg)** |
|---|---|---|---|---|---|---|---|
| Average | 38 | 7 | 27 | 8.3 | 27 | 13 | 1775 |
| Standard deviation | 21 | 1 | 35 | 8.3 | 26 | 11 | 4103 |
| Coefficient of variation | 56% | 10% | 1.3% | 1.0% | 1.0% | 0.84% | 231% |
| Median | 31 | 7 | 16 | 7.9 | 15 | 12 | 14 |
| Number of values | 18 | 7 | 15 | 6.0 | 9.0 | 12 | 10 |
| Minimum value | 13 | 5.5 | 1.3 | 0.035 | 6.5 | 4.2 | $5x10^{-03}$ |
| Maximum value | 87 | 7.5 | 119 | 20 | 80 | 45 | 13,100 |

**LEGEND:** Summary of kinetic parameters for other reductases reported in the literature (upper table). The numbers in parentheses are the reported $K_d$ or $K_M$ or specific activity values. The lower table summarizes the average, media, maximum, and minimum values reported in the upper table. Citations refer to the citation list in the published manuscript.

## FIGURES

### Figure S1. Residue numbering for Mms6 and mutants

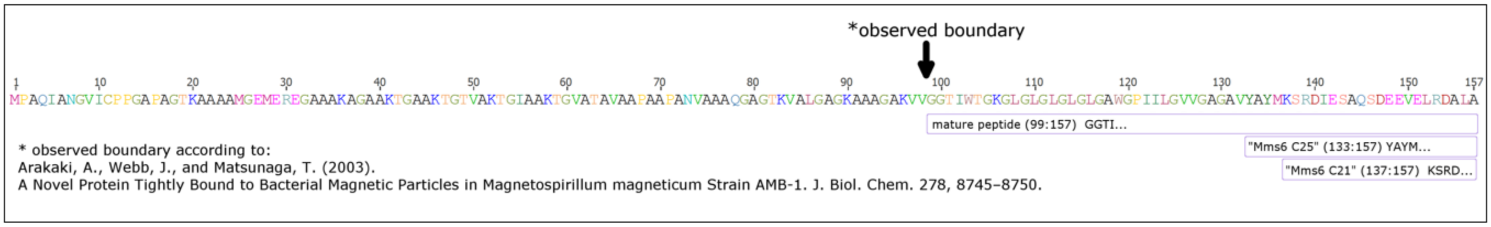


**LEGEND:** The numbering scheme for Mms6 residues relative to the translation of the complete gene sequence.

### Figure S2. Hydropathy plots of Mms6 and its sequence variants used in this work.

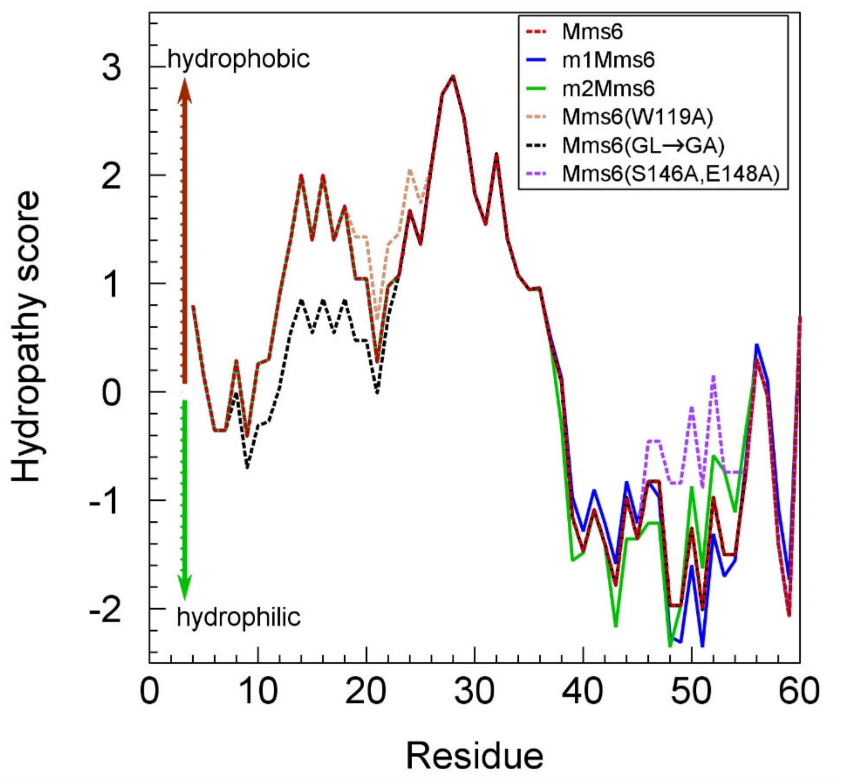


**LEGEND:** Hydropathy plots were obtained using the Web tool: ProtScale (on the ExPASy Server) http://expasy.org/tools/protscale.html. Parameter settings were: window size = 7, Relative weight of the window edges = 100%, Choices in modeling were: Weight variation model = linear, and normalized scale = no.

**Figure S3. The effects of C-terminal sequence scrambles on the ability of Mms6 to form micelles.**

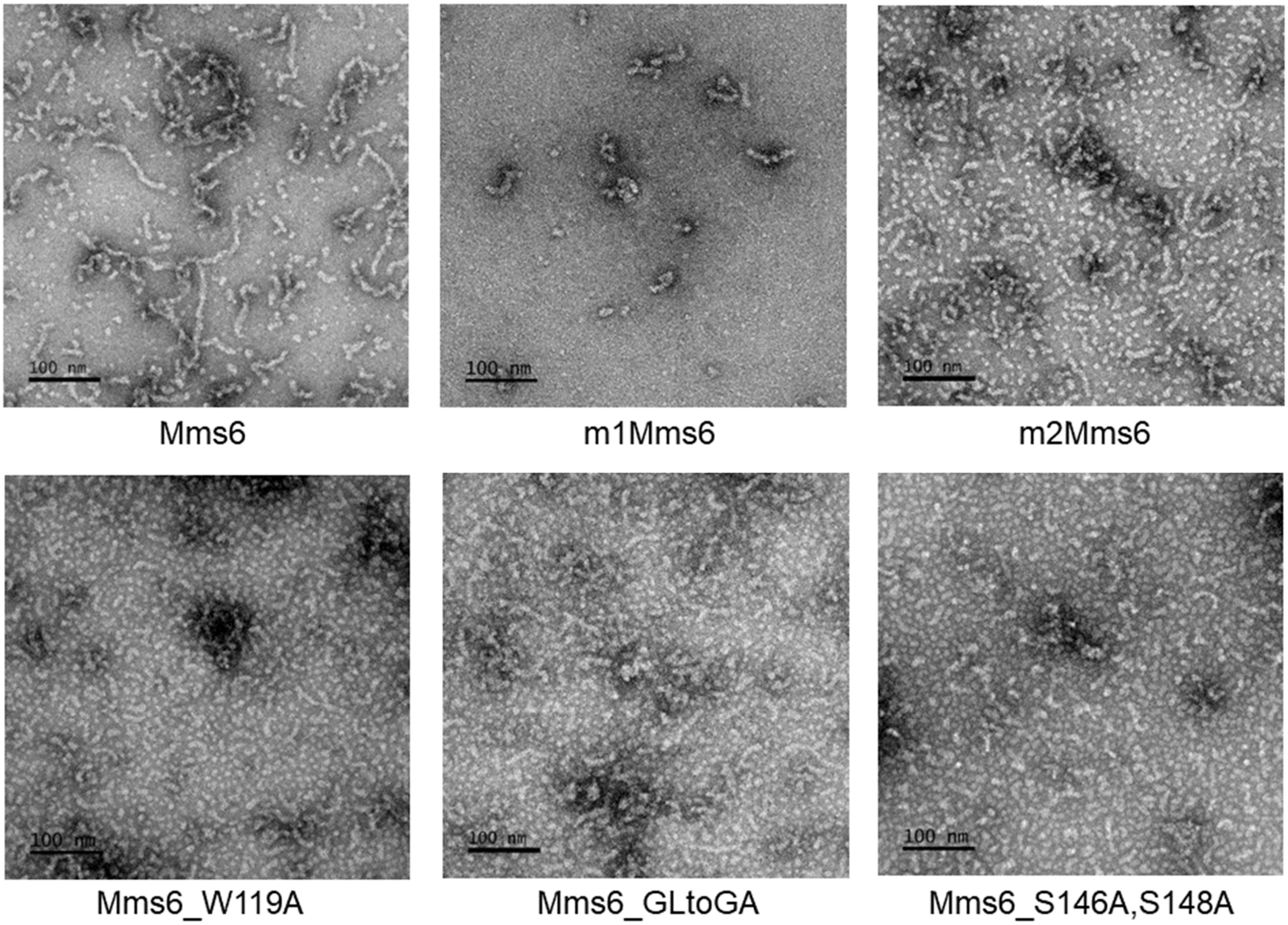


**LEGEND:** Morphologies of Mms6 mutants observed in TEM images. All images are shown at the same resolution (100 nm scale bar). Note that the m1Mms6 protein preparation was at a lower concentration than others but includes the same characteristic in all images of micelles frequently in strings. The concentration of protein in the m1Mms6 preparation was much lower than for the other preparations, which resulted in a sparser distribution of particles.

**Figure S4. $K_M$ estimates for FAD and NADH and a comparison between aerobic and anaerobic conditions.**

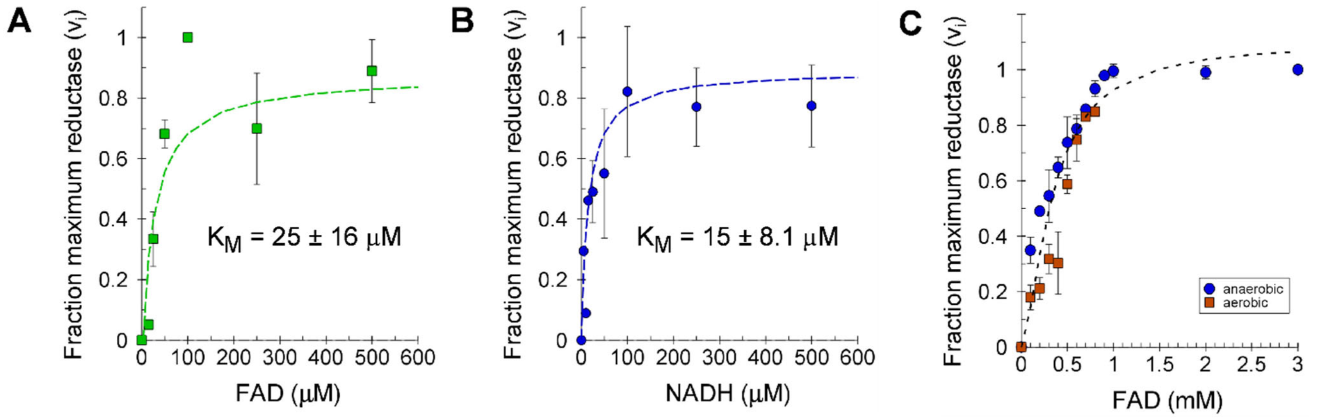


**LEGEND:** Reductase activity (initial velocity) was determined under aerobic conditions over a range of **(A)** FAD concentrations and **(B)** NADH concentrations. Calculated $K_M$ values are shown. These are considered rough estimates as the signal was low, the errors are large and very few points have contributed to the fit. This is reflected by the goodness of fit values (A; $r^2$ = 0.81, standard error of the estimate = 0.17; B: $r^2$ = 0.84, standard error of the estimate = 0.15). **(C)** In a separate set of experiments, the reductase activity at various concentrations of FAD was compared under aerobic and anerobic conditions

**Figure S5. The effect of hemin on Mms6 reductase activity.**

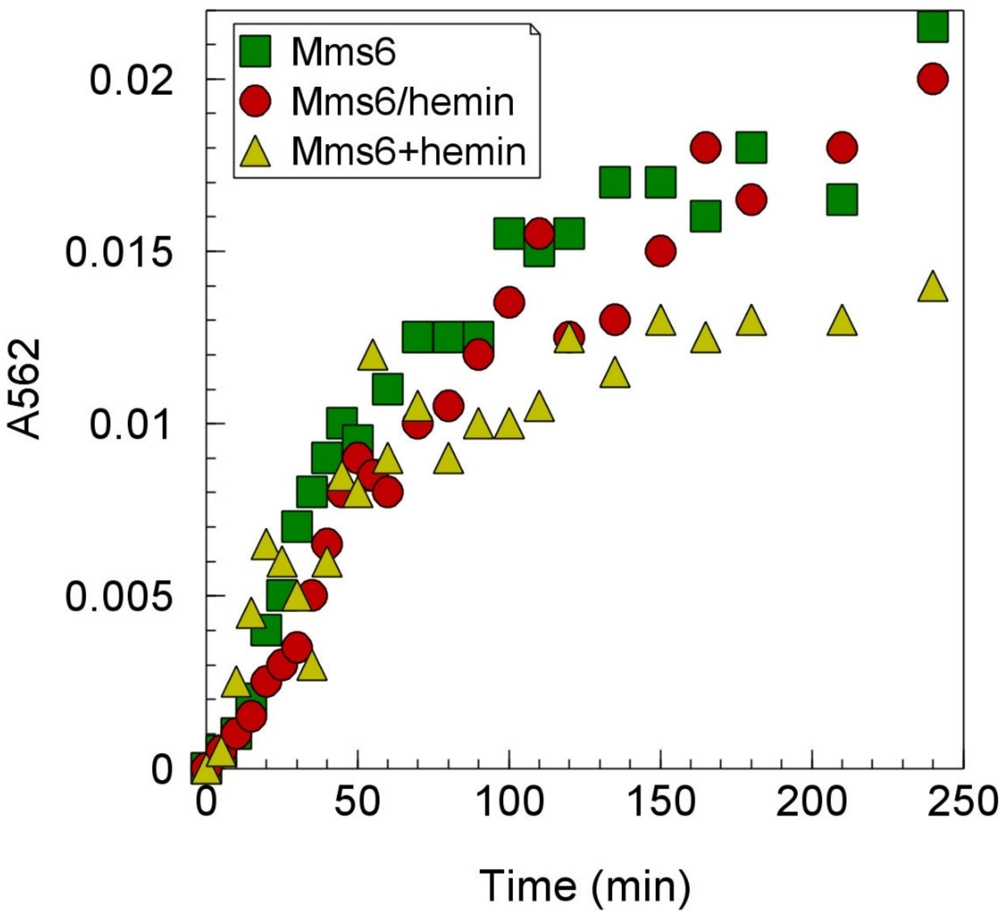


**LEGEND:** Freshly prepared Mms6 (45 µM) in Buffer A (20 mM Tris, 100 mM KCl, 0.05% (v/v) NP-40, 10 mM 2-mercapto-ethanol, 0.2 mM PMSF, pH 7.9) with 7M urea, 200 mM imidazole was dialyzed at 4 °C against Buffer A with consecutive changes to buffers containing incrementally lower concentrations of urea (4, 2, 1 ,0.5, 0 M). After the urea was removed, the sample was divided into two and dialyzed with or without 0.4 mM hemin over a period of 2 hours followed by dialysis against Buffer A for 14 h with one buffer change. The protein dialyzed against hemin is referred to as Mms6/hemin and the Mms6 dialyzed against Buffer A without hemin was named as Mms6. For the sample Mms6+hemin, 20 µM hemin was added directly before the assay was initiated. Twenty µM Mms6 was assayed for reductase activity in the presence and absence of 20 µM hemin in 20 mM Tris, 100 mM KCl, pH 7.5 with 100 µM each of NADH and FAD and 0.8 mM ferrozine.  Mms6/hemin was assayed under the same conditions but in the absence of additional added hemin.

**Figure S6. Ferric reductase activities of Mms6 and mutants with Ala-substitutions in the C-terminal domain.**

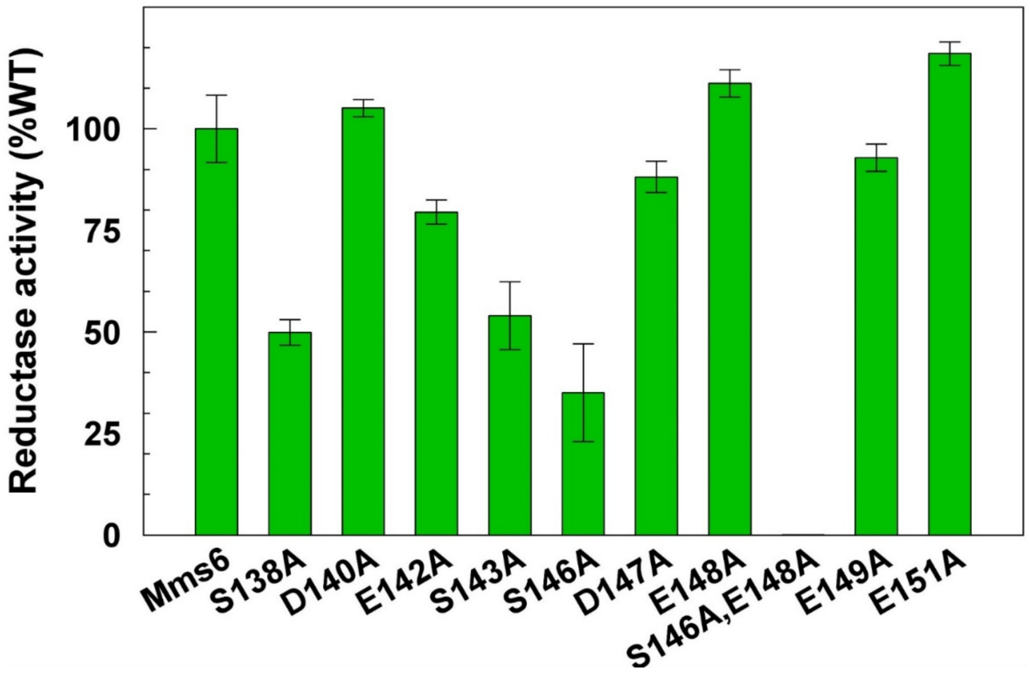


**LEGEND:** The ferric reductase activity of each recombinant protein was determined as described in Materials and Methods with 20 μM protein and 100 μM ferric-citrate.

**Figure S7. Mms6 micelle diameters**

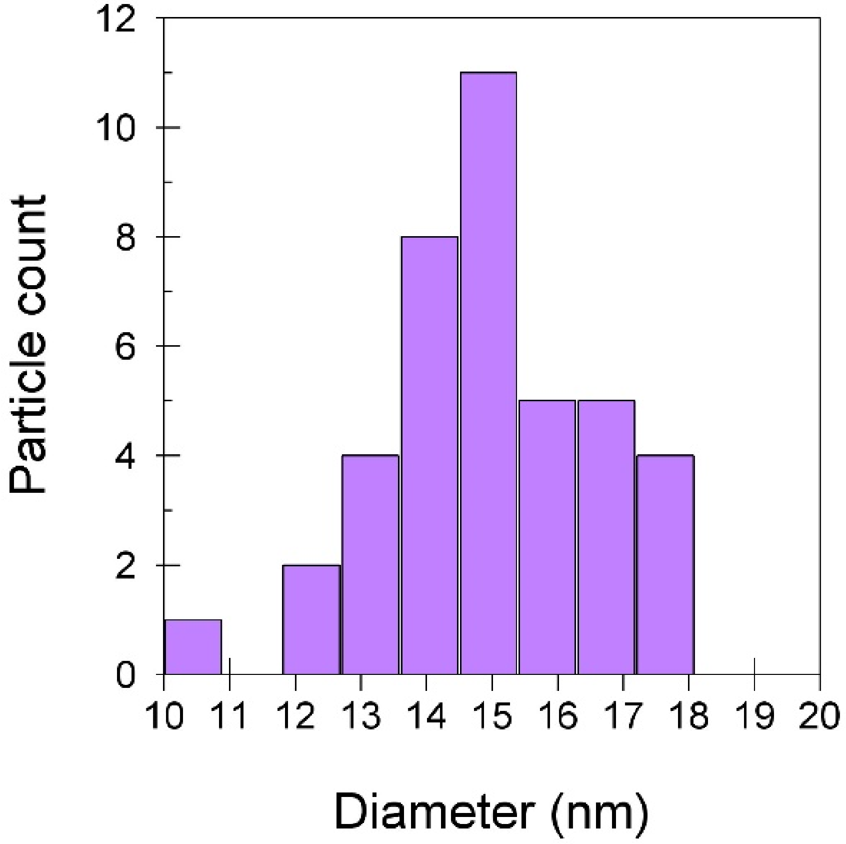


**LEGEND**: Forty spherical particles were measured for their diameter and grouped by size with one group containing all particles within a 1 nm range. Particle counts are shown per group with the bar centered on the average diameter in the group.

**Figure S8. The structure of Mms6 as predicted by a range of modeling algorithms**

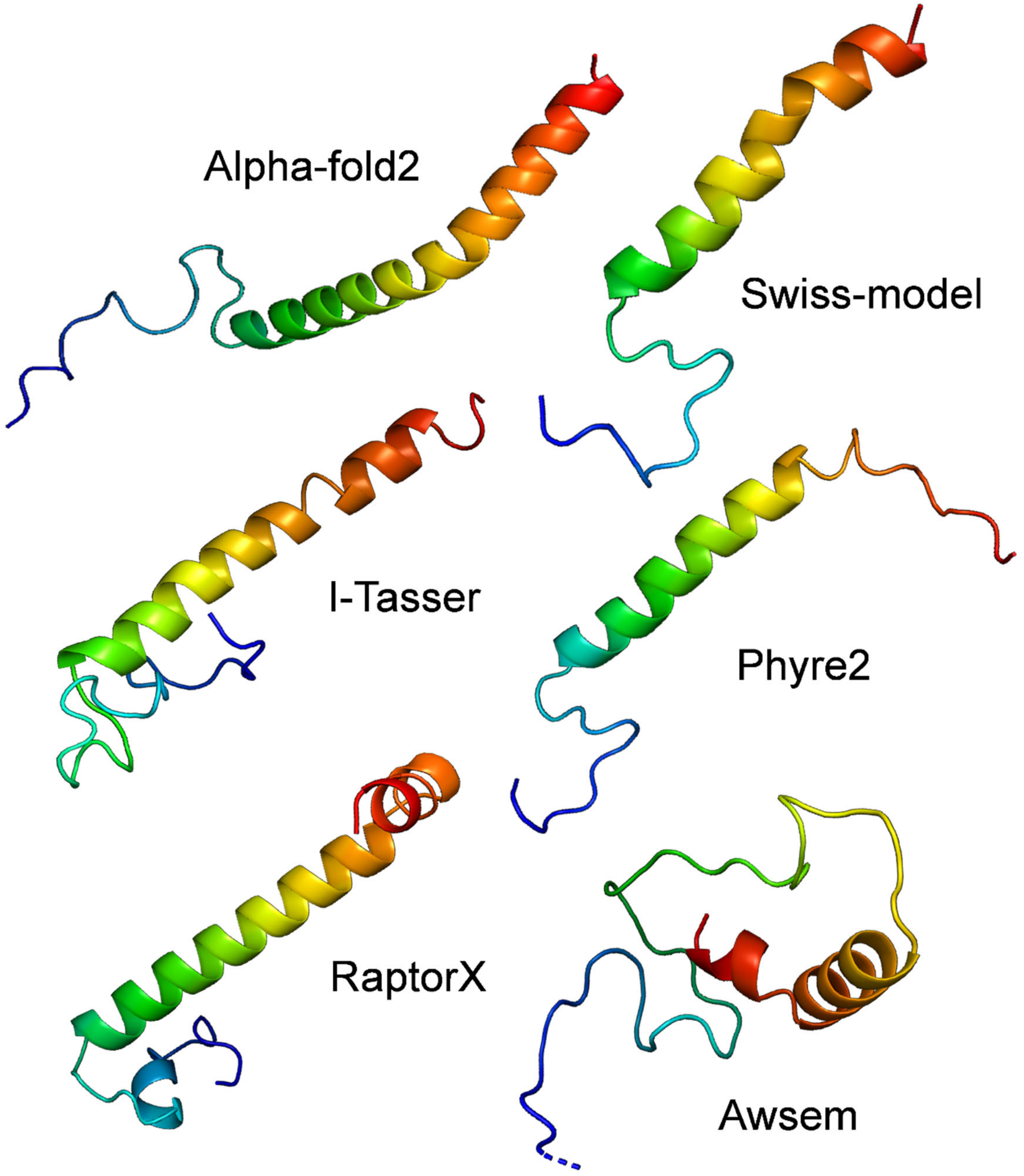


**LEGEND:** All modeling algorithms, except for Awsem, predicted a flexible C-terminal alpha helical domain and a largely unstructured N-terminal domain. All modeling algorithms accessed on 12 June, 2021.

**Figure S9. Predicted quaternary structure of Mms6**

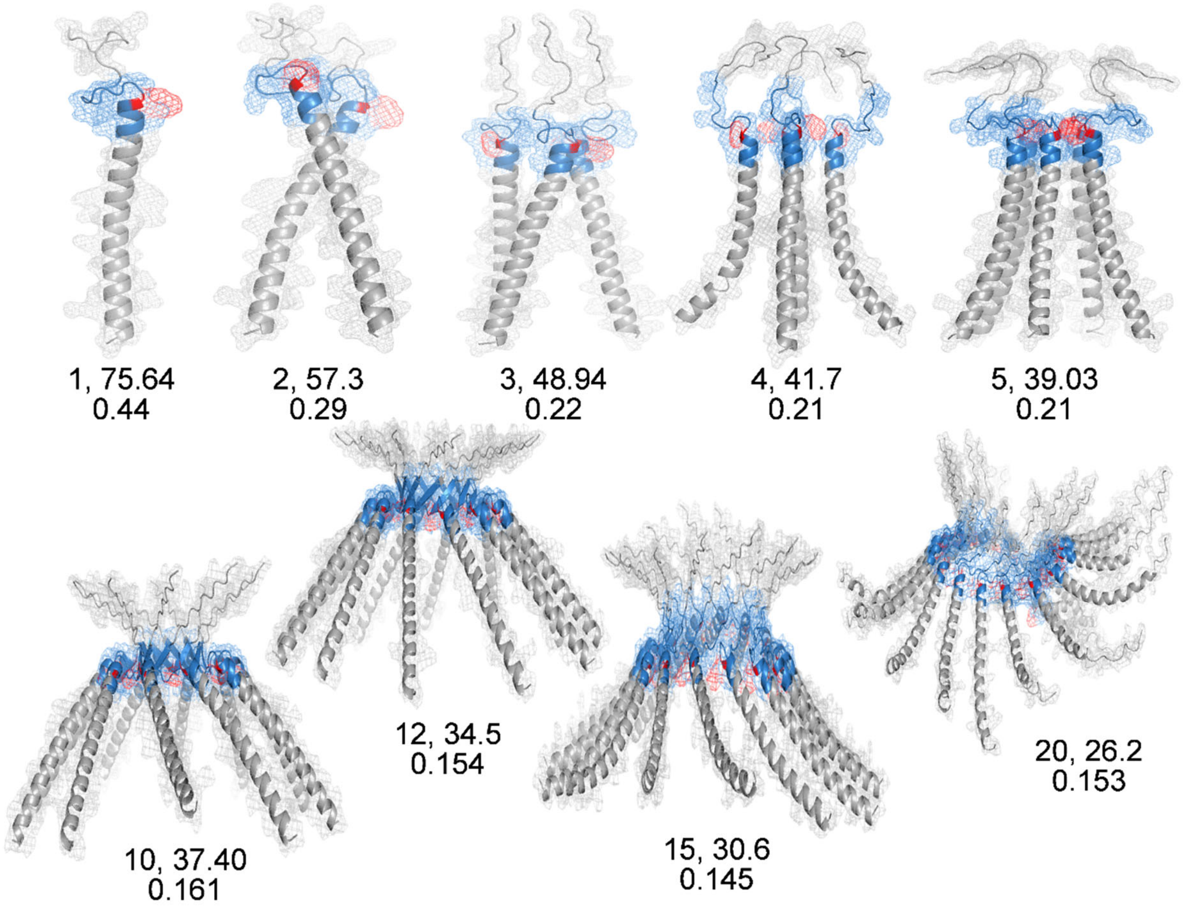


**LEGEND:** The quaternary structure of Mms6 was modeled up to eicosamers by Alphafold2 (accessed on 15 August, 2022). The first row of numbers under each predicted structure identifies the number of Mms6 monomers in the structure and the pLDDT score (predicted local distance difference test, a per-residue confidence metric). A pLDDT of less than 50 predicts likelihood of disorder. The number in the second row is the pTM (predicted template modeling) score, which reflects the level of confidence in the overall predicted structure.

**Figure S10. Identification of the Trp that changes in its hydrophobic environment when Mms6 associates with bicelles**.

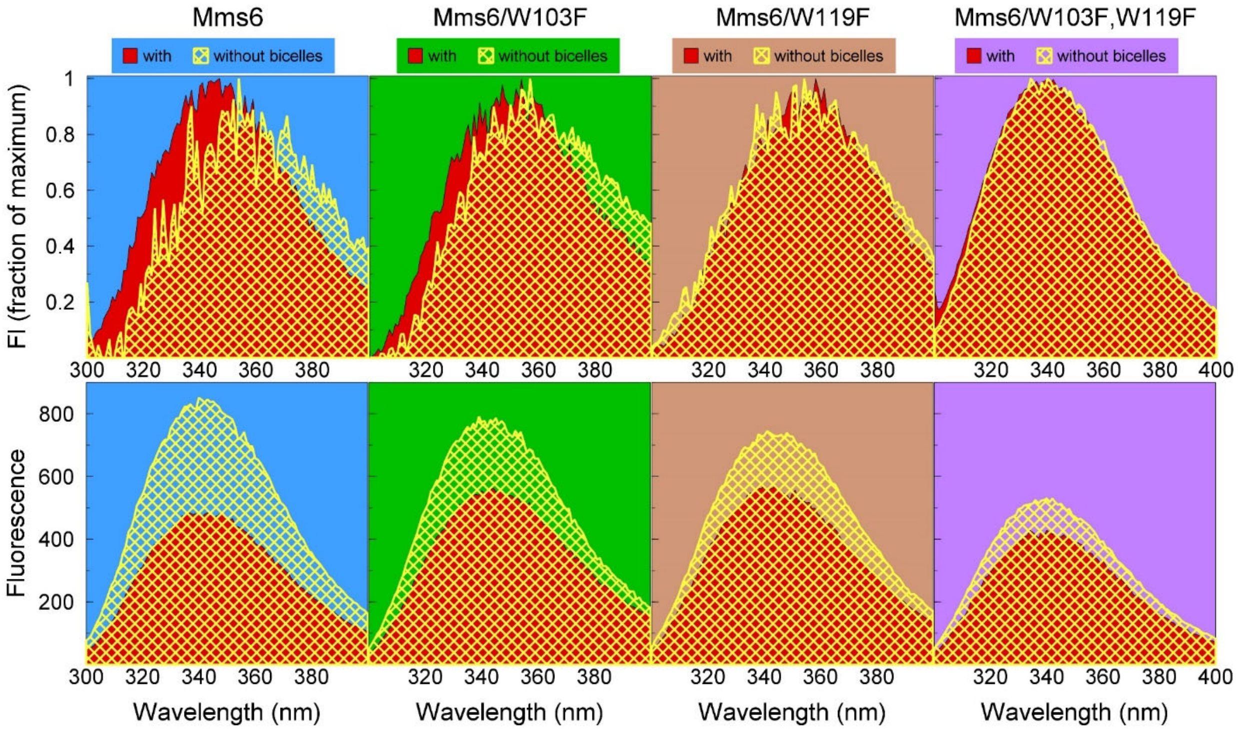


**LEGEND:** Mms6 and the mutant proteins Mms6/W103F, Mms6/W119F and Mms6W103F,W109F at 20 μM were each incorporated into 25 mM q=1 DMPC/DHPC bicelles in a buffer of 20 mM Tris, 100 mM KCl, pH 7.5. The preparations were scanned by a Varian, Cary Eclipse (medium scan rate, PMT detector set at 680V) over the wavelength range 300 to 400 nm in quartz cuvettes with an excitation wavelength of 290 nm. The slit width for both excitation and emission was 5 nm. These results show that the fluorescence shift is lost with W119F but not with W103F.
